# Editorial: “The Host-Microbiome Interplay in Colorectal Cancer”

**DOI:** 10.3389/fcimb.2022.906719

**Published:** 2022-06-08

**Authors:** Laure Campillo-Gimenez, Ye Yang, Clara G. De Los Reyes-Gavilan, Tadahide Izumi

**Affiliations:** ^1^Department of Pathology, University of California San Diego, La Jolla, CA, United States; ^2^Department of Medicine, University of Florida, Gainesville, FL, United States; ^3^Department of Microbiology and Biochemistry of Dairy Products, Instituto de Productos Lacteos de Asturias, Consejo Superior de Investigaciones Cientificas (IPLA-CSIC), Villaviciosa, Asturias, Spain; ^4^Department of Toxicology and Cancer Biology, University of Kentucky, Lexington, KY, United States

**Keywords:** colorectal cancer, microbiome dysbiosis, oncomicrobes, virulence factors, immune memory, IEC, inflammation

## Introduction

Colorectal cancer (CRC) is the third most commonly diagnosed cancer in male and the second in female, according to the World Health Organization. The risk factors to develop CRC include genetic, age, lifestyle/environmental factors (smoking, diet) and obesity; however, the exact cause of CRC is still unknown. Recent advances place the gut microbiome as a key player in the CRC development and progression as well as on the success for the disease treatment. In fact, multi-cohort studies involving geographically diverse populations have revealed distinct and reproducible changes in the microbiota composition in CRC patients and along CRC progression compared to non-CRC controls (Thomas et al., 2019; Wirbel et al., 2019; Yachida et al., 2019). Mechanistically, oncogenic properties of specific bacteria (so called oncomicrobes) have been attributed to bacterial toxins or virulence factors (Lopez et al., 2021). For example, host cell-DNA binding of the secondary metabolite colibactin produced by *pks^+^ Eschericia coli* induces DNA damage and consequently mutagenesis in intestinal epithelial cells (IECs) (Nougayrède et al., 2006; Pleguezuelos-Manzano et al., 2020) that promote tumorigenesis (Arthur et al., 2012). Enterotoxigenic *Bacteroides fragilis* strains producing the BF toxin can trigger a cascade of signaling pathways that lead to chronic intestinal inflammation (Th-17-dependent colitis), tissue injury and CRC tumorigenesis (Wu et al., 2009; Thiele Orberg et al., 2017; Cheng et al., 2020). *Fusobacterium nucleatum*, a bacterial species often found enriched in CRC cases, can induce oncogenic inflammatory responses and attenuate anti-tumor immunity *via* expressing FadA and Fap2 adhesins (Rubinstein et al., 2013; Kostic et al., 2014; Gur et al., 2015).

In this context, this Research Topic aimed to highlight original manuscripts identifying new microbes with oncogenic properties or new cellular mechanisms associated to CRC initiation, promotion and development that could be used as novel tools for the prevention or treatment of CRC.

## New Insights in Cellular Mechanisms Associated to CRC

IECs are key element for maintaining the balance between the microbiota and the host and need to be constantly replenished from the stem cell niche to ensure intestinal barrier integrity. Dysbiosis involving the enrichment in pathogenic species leading to intestinal barrier disruption and inflammation is known as one of the hallmark of CRC (Peterson and Artis, 2014).

In their review, Kendong et al. elaborated that gut dysbiosis, influenced by environmental factors such as diet, could affect the genome, metabolome and immunome as a mechanism of intestinal barrier dysfunction in early-onset CRC. In their original research, Taddese et al. demonstrated for the first time the capacity of *Streptococcus gallolyticus* (and other bacteria) secretome (ensemble of bacterial factors) to increase the aryl-hydrocarbon receptor-dependent expression of CYP1, along with an increased formation of DNA damage in IECs. CPY1 is a biotransformation enzyme oxidizing or hydrolyzing toxic substrates into more reactive intermediates. Therefore, induction of CYP1 by *S. gallolyticus* may result in the accumulation of toxic intermediates leading to CRC carcinogenesis.

Chronic inflammation is also an established risk factor for CRC, as patients with inflammatory bowel diseases have a higher risk than the general population to develop CRC. Dysbiosis or specific oncomicrobes like enterotoxigenic *B. fragilis* can trigger inflammation through the recognition of bacterial antigens by pattern recognition receptors (Wong and Yu, 2019). Especially, Privitera et al. reviewed the role of the “inflammasome-microbiome axis” in the initiation and development of CRC, with a specific focus on the gasdermin family proteins linked to pyroptosis. This review brings to light an emerging area of research and highlights the complexity of context-dependent microbiome influence on inflammasome signaling and CRC pathogenesis. In order to evaluate the role of inflammation in the initiation of CRC, Sobhani et al. presented an interesting case study of monozygotic twin sisters: one suffering from an advanced CRC, and the other one showing two adenomatous polyps with no sign of progression towards CRC, excluding genetics as a risk factor for CRC. An enrichment of virulent bacteria encompassing *E. coli*, and species from *Shigella*, *Clostridium* and *Streptococcus* was observed in the stools from the CRC-twin only. Indeed, it was estimated that about 1% of all bacterial DNA sequencing reads encoded virulent toxin sequences, constituting the main difference between the CRC-suffering and non-CRC twin sisters. The overexpression of pathogenic bacteria was associated with a higher pro-inflammatory cytokine environment (IL-6, TNF-α, CXCL1 expression) and immune cell infiltrate (mainly monocytes, neutrophils and Th1/Th17 cells) within the tumor. These findings suggest that virulent toxins produced by pathogenic bacteria may be involved in the inflammation-mediated carcinogenesis.

## Use of Microbiome as a Tool for Diagnosis, Prevention and Treatment of CRC

Given the key role of the microbiome (bacteria as well as viruses and fungi) in gut homeostasis and its strong link to CRC development, one of the current challenges is to identify biomarkers for early CRC diagnosis and prognostic in order to either prevent CRC development or promote effective treatments. Thus, one axis of research has been to determine the earliest microbiome composition associated with the first signs of CRC lesions (colorectal adenoma) (Wong and Yu, 2019). In their original research, Gao et al. explored the microbiome composition, including bacteriome and virome, of 71 CRC patients, 63 patients with detected colorectal adenoma and 91 healthy donors. They found an enrichment of several particular bacterial species as well as bacteriophages at different stages of the disease (adenoma vs CRC patients). Moreover, mutations in the KRAS oncogene were identified specifically among CRC genomes that were associated with particular types of bacteriophages suggesting their potential role as driver microbes. These findings may help develop diagnosis specifically focusing on the CRC risk factors. With the same purpose, Yao et al. explored the existing datasets for bacterial species showing differential abundance in the stools of CRC patients and non-CRC individuals. This led to a panel of 5 bacteria consisting of *Prevotella copri*, *Gemella morbillorum*, *Parvimonas micra*, *Cetobacterium somerae* and *Pasteurella stomatis*, which showed higher diagnostic ability for CRC when combined with the fecal immunochemical test. The study provides novel knowledge on how the microbiota could be leveraged for non-invasive detection of CRC.

Determining the gut microbiome composition related to CRC, and understanding how the environmental factors – especially the diet – affect it or how microbes can transmit a particular phenotype may led to the development of new therapeutic strategies. Microbiome editing (or the art of manipulating the microbiome) emerged as a credible approach to limit the impact of microbes on CRC (Zhu et al., 2019). However, in their mini-review, Campillo-Gimenez et al. argued that success of microbiome editing to prevent or treat CRC cannot be achieved without considering the pre-existing immunological memory to a specific microbiome that may bias colonization with new species and impair attempts to re-equilibrate host responses.

## Conclusion

It is now clear that the gut microbiota plays a role in every step of CRC pathogenesis. With the increasing interest in understanding the involvement of the gut microbiota in CRC development and treatment, we can expect to see more refined microbiota-based approaches emerging for CRC early diagnosis, prevention and therapy.

## Author Contributions

L. Campillo-Gimenez built and led the team of Correspondence for the editorial work of this special issue. All authors listed have made a substantial, direct, and intellectual contribution to the work and approved it for publication.

## Funding

The work of editors related to this research topic received financial support from: Spanish project RTI2018-098288-B-I00 financed by MCIN/AEI/10.13039/501100011033/FEDER “Una manera de hacer Europa” (GC), NIH R01-AI079145 (C-GL) and NIH R03-CA249111 (IT).

## Conflict of Interest

The authors declare that the research was conducted in the absence of any commercial or financial relationship that could construed as a potential conflict of interest.

## Publisher’s Note

All claims expressed in this article are solely those of the authors and do not necessarily represent those of their affiliated organizations, or those of the publisher, the editors and the reviewers. Any product that may be evaluated in this article, or claim that may be made by its manufacturer, is not guaranteed or endorsed by the publisher.
